# Risk Factors Associated with Unplanned Hospitalization Among Long-Term Care Facility Residents: A Retrospective Study in Central Taiwan

**DOI:** 10.3390/healthcare12202069

**Published:** 2024-10-17

**Authors:** Chiu-Hsiang Lee, Yu-An Chen, Chiu-Ming Yang, Kuang-Hua Huang, Tung-Han Tsai, Yuanmay Chang, Shwn-Huey Shieh

**Affiliations:** 1 School of Nursing, Chung Shan Medical University, Taichung 402, Taiwan; csha528@csmu.edu.tw; 2 Department of Nursing, Chung Shan Medical University Hospital, Taichung 402, Taiwan; 3 Department of Education, Taichung Veterans General Hospital, Taichung 407, Taiwan; b101104014@tmu.edu.tw; 4 Department of Health Services Administration, China Medical University, Taichung 406, Taiwan; sanann861109@gmail.com (C.-M.Y.); khhuang@mail.cmu.edu.tw (K.-H.H.); dondon0525@gmail.com (T.-H.T.); 5 Institute of Long-Term Care, Mackay Medical College, New Taipei City 252, Taiwan; r000085@mmc.edu.tw; 6 Department of Nursing, China Medical University Hospital, Taichung 406, Taiwan; 7 Department of Nursing, Asia University, Taichung 413, Taiwan

**Keywords:** hospitalization, long-term care, nursing home, residential facilities, risk factors

## Abstract

Most residents of long-term care facilities (LTCFs) are patients with chronic diseases requiring long-term care. Unplanned hospitalization of older and frailer residents from LTCFs reduces their mobility and increases the number of infections, complications, and falls that might lead to severe disability or death. This study aimed to identify the critical risk factors associated with unplanned hospitalization among LTCF residents in Taiwan, providing insights that could inform better care practices in similar settings globally. A retrospective study was conducted using inpatient data from a medical center in central Taiwan, covering the period from 2011 to 2019. A total of 1220 LTCF residents were matched with general patients using propensity score matching. Multiple logistic regression analyses were performed to identify factors associated with unplanned hospitalization, controlling for relevant variables. LTCF residents had a significantly higher risk of unplanned hospitalization compared to general patients (OR = 1.44, 95% CI = 1.21–1.73). Key risk factors included advanced age (≥85 years, OR = 1.25, 95% CI = 1.02–1.54), the presence of comorbidities such as diabetes (OR = 1.17, 95% CI = 1.03–1.33) and renal failure (OR = 1.63, 95% CI = 1.42–1.86), high fall risk (OR = 2.67, 95% CI = 2.30–3.10), and being bedridden (OR = 6.55, 95% CI = 5.48–7.85). The presence of a tracheostomy tube also significantly increased hospitalization risk (OR = 1.73, 95% CI = 1.15–2.59). LTCF residents are at a higher risk of unplanned hospitalization, particularly those with specific comorbidities, physical limitations, and indwelling medical devices. These findings underscore the need for targeted interventions to manage these risks, potentially improving care outcomes for LTCF residents globally.

## 1. Introduction

Most residents of long-term care facilities (LTCFs) are patients with chronic diseases requiring long-term care or patients who must continue receiving medical care after being discharged from the hospital. Unplanned hospitalization of older and frailer residents from LTCFs reduces their mobility and increases the number of infections, complications, and falls that might lead to severe disability or death; thus, unplanned hospitalizations negatively impact the health of residents [1] and serve as a key quality indicator for these facilities. Reducing their incidence can enhance care quality and lower healthcare costs [2]. Most of them are older adults with chronic conditions such as hypertension, stroke, and diabetes, placing them at higher risk for respiratory and heart-related diseases [3]. Studies show that nearly all residents have multiple comorbidities, and the more severe these are, the higher the rate of hospitalizations. In addition, severe trauma caused by falls and indwelling tubes is a common cause of hospitalization [4,5,6].

Infections are a leading cause of unplanned hospitalizations in these settings [5,7]. Most residents have limited mobility and require prolonged bed rest, which is likely to cause pressure ulcers and lead to a higher risk of infection [8]. According to several European studies, the incidence of pressure ulcers in LTCFs ranges from 4.03% to 13.4% [9,10,11,12], while in the United States, it is reported between 8.8% and 9.3% [13]. Older adults aged 65 years are at a high risk of developing pressure ulcers as a result of malnutrition, being underweight or overweight, and having diabetes, renal failure, blood disorders (e.g., anemia or leukemia), or other health problems [12,14,15,16].

The use of urinary catheters, tracheostomy tubes, and nasogastric tubes further heightens the risk of hospitalization by increasing susceptibility to infections [17,18,19]. Tracheostomy and nasogastric tubes are known contributors to pneumonia, a significant cause of hospital admissions [20,21]. In addition, urinary catheters are a common cause of urinary tract infections. More severe urinary tract infections can result in bacteremia or sepsis [22,23].

Related research in Asian countries regarding the aforementioned is rare. An early study in Hong Kong reported that 9.7% of nursing home residents had at least one emergency department (ED) visit, and 24.8% had at least one hospital admission [24]. A one-year cohort study on dementia patients residing in Taiwanese nursing homes found an incidence density of 1722 ED visits per 1000 person-years [25]. Additionally, an investigation of a nursing home in northern Taiwan revealed that 64.3% of residents experienced at least one hospital admission [26]. As mentioned above, the issue should be noticed, particularly in Taiwan, where the incidence is notably high. Few studies have examined this issue, with a notable lack of large, rigorous investigations. This study analyzes the occurrence and factors influencing unplanned hospitalization in LTCF residents in Taiwan via a robust statistical matching method.

## 2. Materials and Methods

### 2.1. Data Source

This study conducted a secondary data analysis by using the inpatient data from 2011 to 2019 of a medical center in central Taiwan as raw data. The data information included the patient’s basic characteristics, comorbidities, physical restraints, fall and pressure ulcer risk assessment results, and tube placement records.

### 2.2. Study Subjects

Inpatients from 2011 to 2019 were used as the research population, and they were divided into LTCF residents and general patients on the basis of whether they were from an LTCF. The LTCF patients were considered the case group. To prevent deviations in the research results and selection bias, this study used propensity score matching (PSM) according to age, sex, diabetes diagnosis, hypertension diagnosis, heart disease diagnosis, and renal failure diagnosis. The PSM is a statistical matching technique that is available to reduce potential confounding caused by unbalanced covariates in non-experimental settings. The propensity score represents the probability calculated through a logistic regression model. This probability is assigned to LTCF residents based on specific characteristics, and it can help reduce or eliminate selection bias when comparing LTCF residents with general patients. The PSM was used to establish a control group of general patients who were matched with LTCF patients for each year at a ratio of 1:5. After the matching was completed, the data of 1220 LTCF patients were included as the case group and those of 6100 general patients were included as the control group.

### 2.3. Study Design

This study was a retrospective study and set unplanned hospitalization as the dependent variable. Unplanned hospitalization was defined as an unexpected, nonselective urgent or emergent hospitalization for acute illness or for complications of care in LTCF residents. This study set patient’s basic characteristics (i.e., sex and age), physical assessment results (i.e., diabetes, hypertension, heart disease, renal failure, other comorbidities, BMI, state of consciousness, physical restraint, and activities of daily living), fall and pressure ulcer risk assessment results, and records of tube placement (i.e., nasogastric tube, urinary catheter, and tracheostomy tube) as the control variables. Consciousness status in this study was assessed using the Glasgow Coma Scale (GCS) and categorized into three levels: mild disturbance (scores 13–15), moderate disturbance (scores 9–12), and severe disturbance (scores 3–8). The STRATIFY risk assessment tool was used to evaluate the fall risk in this study, with patients scoring greater than 4 being classified as high fall risk.

### 2.4. Statistical Analysis

SAS 9.4 was used for data processing and a statistical analysis, and *p* < 0.05 was considered significant. Descriptive statistics were used to analyze the sex, age, and physical assessment results (comorbidities, BMI, state of consciousness, physical restraint, and activities of daily living), fall and pressure ulcer risk assessment results, and tube placement of the LTCF patients; the distribution, percentage, mean, and standard deviation of the research samples were determined. Furthermore, the Chi-squared test was conducted to analyze the variable distribution differences between the LTCF and general patients. After the relevant variables were controlled, multiple logistic regression in the Enter mode was used to predict the factors influencing unplanned hospitalization among LTCF residents.

## 3. Results

### 3.1. Baseline Characteristics after Matching

Table 1 shows the distributions of baseline characteristics between LTCF and general patients after matching. In LTCF patients, 55.98% were male, and the average age was 77.82 years. In terms of comorbidities, 40.57% had diabetes, 58.03% had hypertension, 40.49% had heart disease, and 38.69% had renal failure in LTCF patients. As expected, the characteristics of matching variables were similar between the LTCF and general patients (*p* > 0.05), including sex, age, and comorbidities, after matching.

### 3.2. Incidence of Unplanned Hospitalization

As Table 2 shows, the unplanned hospitalization rate of the LTCF patients was 77.54%, which was significantly higher than that of the general patients (*p* < 0.001). Female patients had a higher incident rate than males (*p* = 0.019), and the incident rate increased with the age of the patients (*p* < 0.001). Patients with comorbidities, including diabetes, hypertension, heart disease, or renal failure, had higher rates of hospitalization (*p* < 0.001). In addition, patients with moderate to severe impairment, physical restraints, bedridden status, or higher risks of falls and pressure ulcers had significantly higher rates of hospitalization (*p* < 0.001). Patients with tube placement also had a higher incident rate, including nasogastric, urinary catheter, or tracheostomy tube (*p* < 0.001).

### 3.3. Factors Associated with Unplanned Hospitalization

Table 3 presents the factors influencing the unplanned hospitalization. After the relevant variables were controlled, LTCF patients had a higher risk of being hospitalized (OR = 1.44, 95% CI = 1.21–1.73), compared with general patients. Patients aged ≥85 years had a 1.25 times higher risk of being hospitalized than patients aged <65 (95% CI = 1.02–1.54). In terms of comorbidities, patients with diabetes (OR = 1.17, 95% CI = 1.03–1.33) and renal failure (OR = 1.63, 95% CI = 1.42–1.86) both had a higher risk of being hospitalized. The risk decreased with the BMI (OR = 0.97, 95% CI = 0.96–0.98). Fall risk, activity status, pressure ulcer risk, and tracheostomy tube were factors associated with unplanned hospitalization.

## 4. Discussion

Most LTCF residents experience chronic diseases that may be complicated with disability and dementia. As a result, they must rely on professional caregivers providing them with long-term and complete nursing care on a daily basis. This study discovered that compared with general patients, LTCF patients, particularly those who are older and have comorbidities, have a significantly higher unplanned hospitalization rate; this result aligns with those presented in the literature [3,5]. Diabetes is a common chronic disease among older adults and was reported to increase the risk of developing a disabling disease and severe complications, such as cardiovascular disease, peripheral vascular disease, neuropathy, retinopathy, and renal failure [27]. A study indicated that the elderly living in LTCFs often have health problems, such as cognitive impairment, depression, physical disability, nutritional problems, and repeated infection. Most of them require invasive treatment and care, such as tube placement, hemodialysis, and ventilators, which may affect their physiological indices (e.g., blood sugar and nutrition) and drug management (e.g., insulin and other hypoglycemic agents) as well as increase the difficulty of diabetes care and management. The higher risk of severe diabetes-related complications and multimorbidity also increases the chance of unplanned hospitalization [28].

The results of the present study demonstrate that the placement of nasogastric tubes, urinary catheters, and tracheostomy tubes was more common in LTCF patients than in general inpatients; the differences were significant, and the result concurs with those of other research [5,18,19,29,30]. Indwelling tubes may increase the risk of unplanned hospitalization because of the infections and severe complications frequently caused by tube placement. Patients with indwelling tubes require more medical resources for treatment and care. When multivariable control was employed, only tracheostomy tube placement significantly affected unplanned hospitalization (OR = 1.73, *p* = 0.008). A study discovered that although tracheostomy tube placement is often used to maintain a patent airway in LTCFs, a lack of proper care or the state of individual health after the tracheostomy tube placement can easily lead to life-threatening complications, such as pneumothorax and hypoxia [31]. Long-term placement of an artificial trachea may lead to pressure-induced adverse outcomes, such as oral ulceration, vocal cord damage, or difficulty swallowing. As a result, the risk of nutritional imbalance and wound infection increases [29,30]; the complexity of their diseases is aggravated; and the risk of unplanned hospitalization becomes higher [17,21]. To control the rate of unplanned hospitalization, nurses and nursing assistants of the LTCFs should pay attention to the physiological status and wound care of the catheterized residents to reduce their risks of infection-related complications.

According to the study results, the risk of pressure ulcers was significantly higher among LTCF patients than it was among non-LTCF patients. Compared with LTCF patients with a low pressure ulcer risk, those with a high pressure ulcer risk had a significantly higher risk of unplanned hospitalization; this result aligns with those of other research [9,10,11,12,29,30]. Repeated infection of pressure ulcers can lead to cellulitis, myeloma, and other severe symptoms. As a result, the length of hospitalization after admission may increase, causing the consumption of more medical resources [8]. Pressure ulcers are preventable injuries. Nutritional supplementation, skincare, regular turning, or assistive devices can effectively reduce the incidence of pressure ulcers who are at a high risk of pressure ulcers and can reduce the risk of subsequent infection [8,12,14,15,16].

In terms of the limitations of this study, only the inpatient data of a medical center in central Taiwan were used as raw data for the analyses. Because data may vary with the regions they are obtained from, the study results have tenuous extrapolation and do not represent the actual situations of all medical institutions. This study also used data obtained from a database for analysis, which affected the overall data integrity because the data contained incomplete records and missing values. In addition, this study analyzed only the impact of LTCF patients’ health, nutritional status, and tube placement on hospital transfers. Because the LTCF levels, fee schedules, and non-LTCF patients’ personal factors were not analyzed in this study, the results cannot be used to represent or infer the factors influencing unplanned hospitalization in all LTCFs.

This study identified the risk factors influencing the acute medical hospitalization of LTCF residents. LTCF caregivers should obtain an in-depth understanding of the factors that trigger resident hospitalization and formulate appropriate preventive measures. The government may use the research results as a reference for formulating long-term care quality policies and for training professional caregivers in LTCFs. By doing so, the government can improve the quality of nursing care in LTCFs, reduce the risk of being hospitalized, reduce wasteful use of medical resources, lower unnecessary medical expenses, and increase awareness and affirmation of LTCF care among residents and family members.

## 5. Conclusions

In the present study, when multivariate statistics were controlled for, the unplanned hospitalization rate of LTCF residents was higher than that of general patients. In addition, patients with comorbidities (e.g., diabetes and renal failure) had a higher unplanned hospitalization rate. Patients at a high risk of falls and pressure ulcers or who required prolonged bed rest had higher chances of unplanned hospitalization. Patients with tracheostomy also had a considerably higher chance of unplanned hospitalization.

## Figures and Tables

**Table 1:** Distributions of baseline characteristics after matching.

Variable	Before Matching					After Matching				
	General Patients		LTCF Patients		*p*-Value	General Patients		LTCF Patients		*p*-Value
	N	%	N	%		N	%	N	%	
Total	141,278	100	1220	100		6100	100	1220	100	
Gender ^1^					<0.001					0.992
Female	70,817	50.13	537	44.02		2684	44.00	537	44.02	
Male	70,449	49.87	683	55.98		3416	56.00	683	55.98	
Missing	12									
Age ^1^					<0.001					1.000
Mean ± SD	52.62 ± 24.43		77.82 ± 15.31			76.07 ± 17.74		77.82 ± 15.31		
<65	89,661	63.46	172	14.1		860	14.10	172	14.10	
65~74	25,323	17.92	206	16.89		1030	16.89	206	16.89	
75~84	16,034	11.35	407	33.36		2035	33.36	407	33.36	
≥85	10,260	7.26	435	35.66		2175	35.66	435	35.66	
Diabetes ^1^					<0.001					1.000
No	117,643	83.27	725	59.43		3625	59.43	725	59.43	
Yes	23,635	16.73	495	40.57		2475	40.57	495	40.57	
Hypertension ^1^					<0.001					0.916
No	101,128	71.58	512	41.97		2550	41.80	512	41.97	
Yes	40,150	28.42	708	58.03		3550	58.20	708	58.03	
Heart disease ^1^					<0.001					0.992
No	120,456	85.26	726	59.51		3631	59.52	726	59.51	
Yes	20,822	14.74	494	40.49		2469	40.48	494	40.49	
Renal failure ^1^					<0.001					1.000
No	122,784	86.91	748	61.31		3740	61.31	748	61.31	
Yes	18,494	13.09	472	38.69		2360	38.69	472	38.69	
BMI	14,120		1211		<0.001	6087		1211		<0.001
Mean ± SD	22.94 ± 5.34		21.70 ± 4.43			23.35 ± 4.50		21.70 ± 4.43		
Missing	158		9			13		9		
Consciousness					<0.001					<0.001
Mild disturbance	123,063	87.11	610	50		5216	85.51	610	50.00	
Moderate disturbance	4372	3.09	299	24.51		438	7.18	299	24.51	
Severe disturbance	13,843	9.8	311	25.49		446	7.31	311	25.49	
Physical restraint					<0.001					0.011
No	140,297	99.31	1187	97.3		6000	98.36	1187	97.30	
Yes	981	0.69	33	2.7		100	1.64	33	2.70	
Activity					<0.001					<0.001
Can get out of bed	112,365	90.24	385	31.77		4649	78.25	385	31.77	
Can’t get out of bed	12,153	9.76	827	68.23		1292	21.75	827	68.23	
Missing	16,760		8			159		8		
Fall risk					<0.001					<0.001
Low risk	89,509	64.89	91	7.67		1710	28.59	91	7.67	
High risk	48,438	3511	1095	92.33		4272	71.41	1095	92.33	
Missing	3331		34			118		34		
Pressure ulcer risk					<0.001					<0.001
Low risk	137,579	97.57	812	66.72		5666	93.04	812	66.72	
High risk	3421	2.43	405	33.28		424	6.96	405	33.28	
Missing	278		3			10		3		
Indwelling catheter										
Nasogastric tube					<0.001					<0.001
No	133,624	94.58	861	70.57		5435	89.10	861	70.57	
Yes	7654	5.42	359	29.43		665	10.90	359	29.43	
Urinary tube					<0.001					<0.001
No	124,717	88.28	928	76.07		5200	85.25	928	76.07	
Yes	16,561	11.72	292	23.93		900	14.75	292	23.93	
Tracheostomy tube					<0.001					0.006
No	138,426	97.98	1158	94.92		5890	96.56	1158	94.92	
Yes	2852	2.02	62	5.08		210	3.44	62	5.08	

^1^ Matched variable.

**Table 2:** Bivariate analysis of unplanned hospitalization.

Variables	Unplanned Hospitalization				
	No		Yes		*p*-Value
	N	%	N	%	
Total	3372	46.07	3948	53.93	
Inpatients					<0.001
General patients	3098	50.79	3002	49.21	
LTCF patients	274	22.46	946	77.54	
Gender					0.019
Female	1434	44.52	1787	55.48	
Male	1938	47.28	2161	52.72	
Age					<0.001
Mean ± SD	72.79 ± 18.07		79.41 ± 16.17		
<65	635	61.53	397	38.47	
65~74	736	59.55	500	40.45	
75~84	1133	46.40	1309	53.60	
≥85	868	33.26	1742	66.74	
Diabetes					<0.001
No	2168	49.84	2182	50.16	
Yes	1204	40.54	1766	59.46	
Hypertension					<0.001
No	1553	50.72	1509	49.28	
Yes	1819	42.72	2439	57.28	
Heart disease					<0.001
No	2292	52.61	2065	47.39	
Yes	1080	36.45	1883	63.55	
Renal failure					<0.001
No	2388	53.21	2100	46.79	
Yes	984	34.75	1848	65.25	
BMI (Mean ± SD)	23.74 ± 4.47		22.51 ± 4.50		<0.001
Consciousness					<0.001
Mild disturbance	3031	52.03	2795	47.97	
Moderate disturbance	192	26.05	545	73.95	
Severe disturbance	149	19.68	608	80.32	
Physical restraint					<0.001
No	3338	46.44	3849	53.56	
Yes	34	25.56	99	74.44	
Activity					<0.001
Can get out of bed	3014	59.87	2020	40.13	
Can’t get out of bed	264	12.46	1855	87.54	
Missing	94		73		
Fall risk					<0.001
Low risk	1311	72.79	490	27.21	
High risk	1985	36.99	3382	63.01	
Missing	76		76		
Pressure ulcer risk					<0.001
Low risk	3278	50.60	3200	49.40	
High risk	85	10.25	744	89.75	
Missing	9		4		
Indwelling catheter					
Nasogastric tube					<0.001
No	3123	49.60	3173	50.40	
Yes	249	24.32	775	75.68	
Urinary catheter					<0.001
No	2939	47.96	3189	52.04	
Yes	433	36.33	759	63.67	
Tracheostomy tube					<0.001
No	3315	47.03	3733	52.97	
Yes	57	20.96	215	79.04	

**Table 3:** Factors associated with unplanned hospitalization.

Variables	Unplanned Hospitalization										
	Unadjusted					Adjusted Model					
	OR	95% CI			*p*-Value	OR	95% CI			*p*-Value	VIF
Inpatients (LTCF vs. General)	3.56	3.09	–	4.11	<0.001	1.44	1.21	–	1.73	<0.001	1.26
Gender (Male vs. Female)	0.90	0.82	–	0.98	0.019	1.06	0.94	–	1.18	0.340	1.03
Age (vs. <65)											1.36
65~74	1.09	0.92	–	1.29	<0.001	0.72	0.58	–	0.90	0.003	
75~84	1.85	1.59	–	2.14	<0.001	0.88	0.72	–	1.07	0.205	
≥85	3.21	2.76	–	3.73	<0.001	1.25	1.02	–	1.54	0.035	
Diabetes (Yes vs. No)	1.46	1.33	–	1.60	<0.001	1.17	1.03	–	1.33	0.019	1.35
Hypertension (Yes vs. No)	1.38	1.26	–	1.52	<0.001	0.84	0.73	–	0.96	0.012	1.45
Heart disease (Yes vs. No)	1.94	1.76	–	2.13	<0.001	1.09	0.95	–	1.25	0.204	1.52
Renal failure (Yes vs. No)	2.14	1.94	–	2.35	<0.001	1.63	1.42	–	1.86	<0.001	1.47
BMI	0.94	0.93	–	0.95	<0.001	0.97	0.96	–	0.98	<0.001	1.10
Consciousness (vs. Mild disturbance)											2.24
Moderate disturbance	3.08	2.59	–	3.66	<0.001	0.61	0.48	–	0.77	<0.001	
Severe disturbance	4.43	3.67	–	5.33	<0.001	0.74	0.53	–	1.04	0.067	
Physical restraint (Yes vs. No)	2.53	1.71	–	3.74	<0.001	1.29	0.81	–	2.04	0.279	1.03
Activity (Can’t get out of bed vs. Can get out of bed)	10.48	9.11	–	12.07	<0.001	6.55	5.48	–	7.85	<0.001	1.74
Fall risk (High risk vs. Low risk)	4.56	4.05	–	5.13	<0.001	2.67	2.30	–	3.10	<0.001	1.36
Pressure ulcer risk (High risk vs. Low risk)	8.97	7.13	–	11.28	<0.001	1.94	1.43	–	2.63	<0.001	1.91
Indwelling Catheter											
Nasogastric tube (Yes vs. No)	3.06	2.63	–	3.56	<0.001	1.18	0.94	–	1.48	0.143	1.66
Urinary catheter (Yes vs. No)	1.62	1.42	–	1.84	<0.001	0.84	0.70	–	1.01	0.064	1.39
Tracheostomy tube (Yes vs. No)	3.35	2.49	–	4.50	<0.001	1.73	1.15	–	2.59	0.008	1.33

## Data Availability

Restrictions apply to the availability of these data. Data were obtained from the Chung Shan Medical University Hospital, Taiwan and are available at https://www.csh.org.tw/ with the permission of the Chung Shan Medical University Hospital, Taiwan.
